# Technical Note: Virtual phantom analyses for preprocessing evaluation and detection of a robust feature set for MRI‐radiomics of the brain

**DOI:** 10.1002/mp.13834

**Published:** 2019-10-08

**Authors:** Marco Bologna, Valentina Corino, Luca Mainardi

**Affiliations:** ^1^ Biosignals, Bioimaging and Bioinformatics Laboratory (B3‐Lab) Department of Electronics, Information and Bioengineering (DEIB) Politecnico di Milano Milan Italy

**Keywords:** features stability, MRI, phantoms, radiomics

## Abstract

**Purpose:**

The purpose of the paper was to use a virtual phantom to identify a set of radiomic features from T1‐weighted and T2‐weighted magnetic resonance imaging (MRI) of the brain which is stable to variations in image acquisition parameters and to evaluate the effect of image preprocessing on radiomic features stability.

**Methods:**

Stability to different sources of variability (time of repetition and echo, voxel size, random noise and intensity non‐uniformity) was evaluated for both T1‐weighted and T2‐weighted MRI images. A set of 107 radiomic features, accounting for shape and size, first order statistics, and textural features was used. Feature stability was quantified using intraclass correlation coefficient (ICC). For each source of variability, stability was evaluated before and after preprocessing (Z‐score normalization, resampling, gaussian filtering and bias field correction). Features that have ICC > 0.75 in all the analysis of variability are selected as stable features. Last, the robust feature sets were tested on images acquired with random simulation parameters to assess their generalizability to unseen conditions.

**Results:**

Preprocessing significantly increased the robustness of radiomic features to the different sources of variability. When preprocessing is applied, a set of 67 and 61 features resulted as stable for T1‐weighted and T2‐wieghted images respectively, over 80% of which were confirmed by the analysis on the images acquired with random simulation parameters.

**Conclusion:**

A set of MRI‐radiomic features, robust to changes in TR/TE/PS/ST, was identified. This set of features may be used in radiomic analyses based on T1‐weighted and T2‐weighted MRI images.

## 1 Introduction

Radiomics is one of the most recent fields in medical image analysis and it consists in the extraction of a large number of features from radiological images such as computed tomography (CT), magnetic resonance imaging (MRI) and positron emission tomography (PET) with the final aim of providing “imaging biomarkers” that can be acquired in an inexpensive and non‐invasive way.^1^ Research on radiomics has already been performed in oncology for different purposes like tumor prognosis,^2^ staging,^3^ and prediction of response to treatment,^4^ often with success.

One of the main limitations of radiomics is that the feature value may depend on several factors (type of scanner, resolution, etc…), making the comparison of results from different studies very difficult. This problem becomes particularly relevant for studies that analyze radiological images collected with different imaging acquisition protocols (retrospective or multi‐center studies). Although it is well known that differences in protocols may reduce the reliability of radiomic features,^5^, ^6^, ^7^, ^8^, ^9^, ^10^, ^11^, ^12^, ^13^ research on association between differences in imaging conditions and radiomic features variability is still ongoing. This is also due to the high number of possible confounding factors (which may also be dependent on the specific imaging technique that is considered) whose exhaustive analysis is not trivial.

Phantoms are useful tools to assess variability to imaging conditions, allowing to evaluate the impact of the different acquisition parameters. In particular, online available phantom datasets (such as the one of Kalendralis et al.^14^ for CT) and virtual phantoms (such as BrainWeb^15^ or SimuBloch^11^ for MRI) can further provide a common ground truth for reliability analyses, increasing the reproducibility of the results. Many phantom‐based variability analyses have been reported in literature,^5^, ^8^, ^10^, ^11^, ^12^, ^16^ most of which focused on CT and PET, with only a few studies related to MRI instead.^11^, ^16^ Moreover, the effect of image preprocessing (like resampling and intensity standardization) on feature stability was never properly quantified for MRI images.

In this study different variability analyses were performed on a virtual phantom (BrainWeb) to identify a set of stable radiomic features from T1‐weighted and T2‐weighted MRI. In particular, the following imaging acquisition parameters and sources of variability were considered: time of repetition (TR), and time of echo (TE); pixel spacing (PS) and slice thickness (ST); intensity non‐uniformity (INU); image noise. The analysis was focused on T1‐weighted and T2‐weighted because they are the most used in the clinical practice and are therefore often considered in radiomics studies.^4^, ^17^ Effect of different preprocessing on features stability was also evaluated, in order to quantify the improvements on features stability.

### 1.A. Virtual phantom and simulated datasets

BrainWeb,^15^ an online available three‐dimensional (3D) virtual phantom was used to simulate the MRI‐scans used for the different variability analyses. The digital phantom was obtained by averaging 27 co‐registered MRI images of real patients, acquired with a 1.5 T MRI scanner.^15^, ^18^ Starting from a digital phantom, BrainWeb allows to perform custom MRI acquisitions, by controlling different parameters such as TR, TE, ST, INU and noise level. T1‐weighted and T2‐weighted MRI were simulated in this work.

### 1.B. Segmentation of the regions of interest

The regions of interest (ROIs) used were obtained directly from the BrainWeb website. In particular, 10 3D ROIs of different sizes representing different tissues (cerebrospinal fluid, gray matter, white matter, fat, muscle, skin, skull, glial matter and connective tissue) were considered. Since the ROIs are very large, to reduce the computational complexity of the radiomic features extraction we used only the 11 central slices of each ROI. Since all the images simulated with BrainWeb shared the same physical space, it was possible to use the same three ROIs for all the MRI acquisitions.

### 1.C. Radiomic features extraction

For each MRI acquisition, radiomic features were extracted from each of the 10 ROIs using Pyradiomics 2.1.0.^19^ A total of 107 3D features were computed for each ROI. Among those, 14 were related to shape and size (SS), 18 referred to first order statistics (FOS), 75 were related to texture. Textural features were computed using five different textural matrices: gray level co‐occurrence matrix (GLCM), gray level run length matrix (GLRLM), gray level size zone matrix (GLSZM), neighborhood gray tone difference matrix (NGTDM) and gray level dependence matrix (GLDM). The default extraction parameters of Pyradiomics were used, except for gray‐values discretization. Instead of the fixed bin size discretization (the default of Pyradiomics), we used an intensity discretization with a fixed bin number, because the fixed bin size discretization may not be the best choice in case of images with arbitrary intensity units such as MRI.^20^ In particular, a 32 bins histogram discretization was used. For a more detailed description of the features used, refer to Pyradiomics documentation.^21^

### 1.D. Stability analyses of radiomic features

To test the stability of the features, four different analyses were performed, covering the possible sources of signal variability in MRI images: (a) Analysis of stability to variations in TR/TE; (b) Analysis of stability to variation of voxel size; (c) Analysis of stability to image noise; (d) Analysis of stability to intensity nonuniformity. The different simulation parameters for the different analysis are reported in Table 1. The ranges of interest for TR/TE, PS and ST were chosen to be similar to the ones that can be encountered in the clinical practice. Noise and inhomogeneities were controlled by setting BrainWeb parameters *noise percentage* and *INU percentage*, that were chosen to be equal or higher to the ones of analogous studies of literature using BrainWeb dataset.^22^, ^23^ The resampling of the images to different resolution was done in MatLab 2018a, since BrainWeb does not allow changes in PS. All the other acquisition parameters (TR, TE, noise level and intensity non‐uniformity) were set directly from BrainWeb.

**Table 1:** Simulation parameters for the datasets used for the stability analyses of the study: stability to changes in repetition and echo time; stability to changes in voxel size; stability to image noise; stability to intensity non‐uniformities; stability to random variation of the simulation parameters.

Stability analyses					
	Analysis #1: TR/TE	Analysis #2: voxel size	Analysis #3: noise	Analysis #4: intensity non‐uniformity	Analysis #5: random variations
Number of images	T1w: 42	T1w: 28	T1w: 10	T1w: 4	T1w: 50
	T2w: 48	T2w: 28	T2w: 10	T2w: 4	T2w: 50
Pulse sequence	Spin echo	Spin echo	Spin echo	Spin echo	Spin echo
Time of repetition (TR)	T1w: 350–650 ms, (50 ms step)	T1w: 500 ms	T1w: 500 ms	T1w: 500 ms	T1w: 350–650 ms, (random)
	T2w: 2000–9000 ms (1000 ms step)	T2w: 6000 ms	T2w: 6000 ms	T2w: 6000 ms	T2w: 2000–9000 ms, (random)
Time of echo (TE)	T1w: 5–15 ms, (2 ms step)	T1w: 9 ms	T1w: 9 ms	T1w: 9 ms	T1w: 5–15 ms, (random)
	T2w: 80–130 ms (10 ms step)	T2w: 100 ms	T2w: 100 ms	T2w: 100 ms	T2w: 80–130 ms, (random)
Slice thickness	1 mm	1–7 mm, (1 mm step)	1 mm	1 mm	1–7 mm, (random)
Pixel spacing	1 mm	1–4 mm, (1 mm step)	1 mm	1 mm	1–4 mm, (random)
Noise percentage	0%	0%	9%	0%	0–9% (random)
Intensity non‐uniformity	None	None	None	3 inhomogeneity field + 1 reference INU%: 40%	Random inhomogeneity field (3 available) INU%: 0–40% (random)

For each feature, stability was assessed using the intraclass correlation coefficient (ICC).^24^ In particular, the type of ICC used was the one to measure agreement in mixed‐effect models, equivalent to the (A,1) model described in McGraw et al.^24^ If a feature has an ICC of 1, it means that the changes in the parameter of interest caused no changes in the features, otherwise the lower the value of ICC the lower the stability of the feature.

Feature stability was evaluated for both the features extracted from the original images and for the features extracted from images that underwent a specific preprocessing which was aimed at reducing the effect of a particular source of variability, namely: (a) To correct for changes in the signal due to different TR/TE, Z‐score normalization^25^ was chosen as intensity standard standardization method. (b) Resampling to common isotropic resolution of 1 mm was adopted to correct variability due to voxel size. Isotropic resampling is preferable to anisotropic resampling because it is a better choice for the calculation of 3D textural features.^20^ (c) Image noise was corrected by applying a gaussian filter (3 × 3 × 3 voxel kernel, σ = 0.5). (d) Bias field correction was obtained through the commonly used N4ITK bias field correction algorithm.^26^ In each analysis, Wilcoxon signed rank tests were used to assessed significant variations in the values of ICC between the original and preprocessed images.

### 1.E. Stable features set identification

The results of the previous analyses were used to select a set of stable features. First, a value of 0.75 for the ICC was used as a lower threshold for stability. The value of 0.75 was chosen in agreement to Koo et al.^27^ Second, the features that, considering the preprocessed images, are stable in all the four analyses are included in the final set of stable features.

To compare the stable feature sets obtained from T1‐weighted and T2‐weighted images the Jaccard index (Jacc) was used:

(1) $$Jacc=\frac{n_{stableT1w\cap T2w}}{n_{stableT1w\cup T2w}}$$

where the numerator of Eq. (1) is the number of features in the intersection of the two stable features sets and the denominator is the number of features in the union of the two sets.

### 1.F. Robustness of stable features set on random simulations

The stability of the selected set features was tested on two datasets (one for each image type) composed by 50 images each. The images in those datasets were simulated using random variations of the analyzed parameters (TR, TE, INU, etc…). Details of the T1‐weighted and T2‐weighted datasets are presented in Table 1. This analysis was used to assess what percentage of the selected stable features are stable even when multiple sources of variability are considered, which is typically the case of the images of the clinical practice.

## 2 Results

### 2.A. Stability analyses of radiomic features

#### 2.A.1. Stability to changes in TR and TE

**Figure 1:**
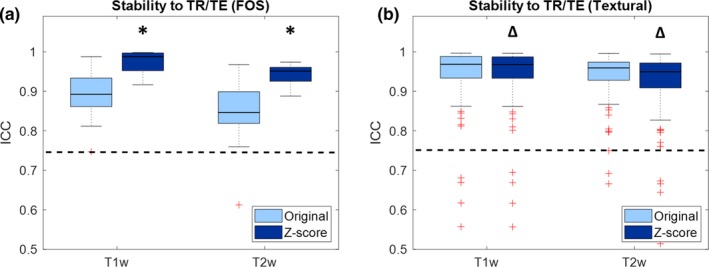
Boxplot representing the intraclass correlation coefficient (ICC) of the radiomic features for stability to variations of repetition and echo time (TR and TE): (a) First order statistics; (b) textural features. Significant differences due to preprocessing are reported with asterisks (ICC increase) or triangles (ICC decrease). The dashed line represents the threshold of stability (ICC = 0.75). [Color figure can be viewed at http://wileyonlinelibrary.com]

Figure 1 shows boxplots representing the values of ICC of the features when the same phantom is acquired with different TR/TE. Values are shown for both T1‐weighted and T2‐weighted images, and for both original and preprocessed images. Only FOS and textural features are considered, since TR/TE variations do not affect the shape and size features.

From Fig. 1 it can be seen that the majority of features are above the threshold of stability. In both T1‐weigthed and T2‐weighted intensity standardization increases ICC values of FOS features (T1w: median increase 0.09 [0.06–0.11], *P* = 7.37*10^−4^; T2w: median increase 0.11 [0.05–0.14], *P* = 8.44*10^−3^) and causes a small but significant decrease in ICC of textural features (T1w: median decrease 5.00*10^−4^ [2.30*10^−5^–1.50*10^−3^], *P* = 3.67*10^‐7^; T2w: median decrease 1.40*10^−4^ [3.21*10^−4^–1.66*10^−3^], *P* = 1.93*10^−11^).

#### 2.A.2. Stability to changes in voxel size

**Figure 2:**
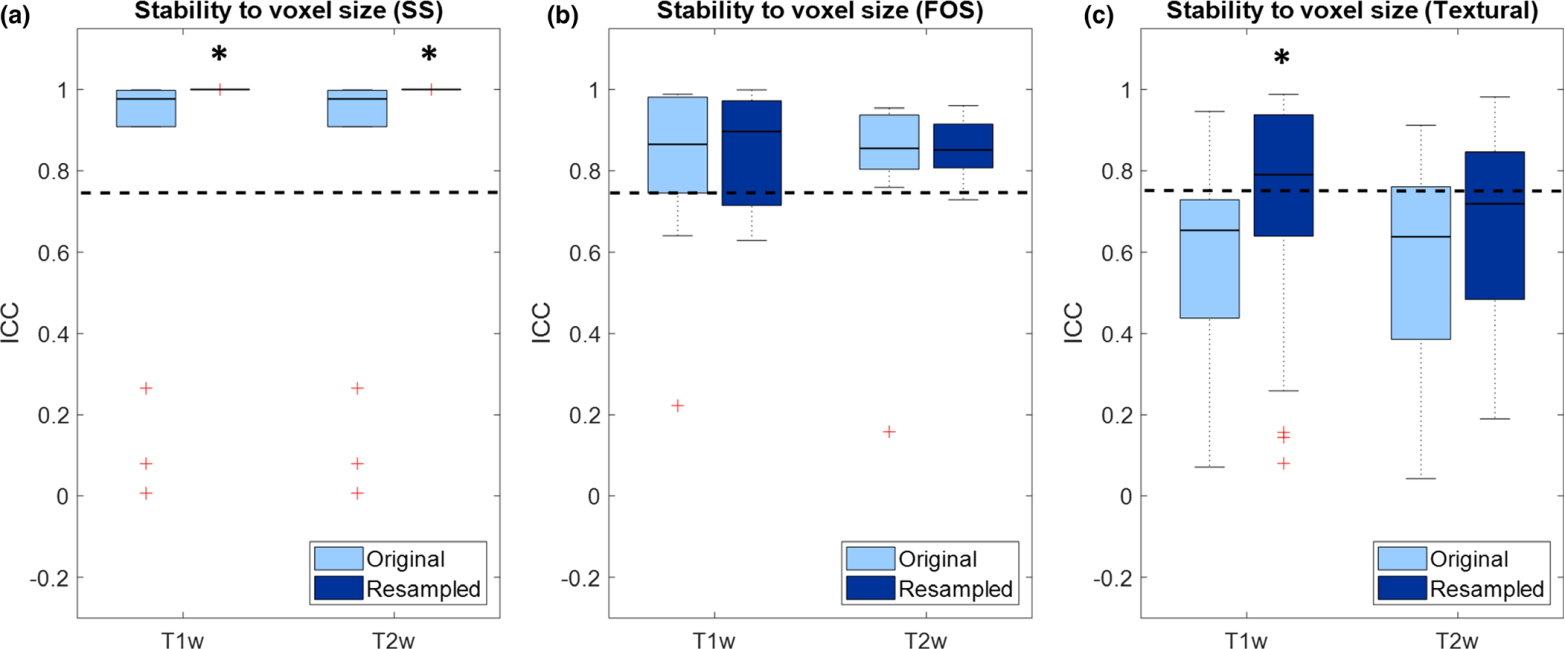
Boxplot representing the intraclass correlation coefficient (ICC) of the radiomic features for stability to variations of voxel size: (a) shape and size; (b) first order statistics; (c) textural features. Significant increase in ICC due to preprocessing are reported with asterisks. The dashed line represents the threshold of stability (ICC = 0.75). [Color figure can be viewed at http://wileyonlinelibrary.com]

Figure 2 shows boxplots representing the values of ICC of the features when the same phantom is acquired with different voxel sizes. Values are shown for both T1‐weighted and T2‐weighted images, and for both original and isotropically resampled images.

From Fig. 2 it can be seen that voxel size has an impact on feature stability, particularly for textural features, whose majority is below the threshold of stability for the original images. In both T1‐weighted and T2‐weighted, resampling to common isotropic resolution significantly increases the stability of shape and size features (T1w/T2w: median increase 0.02 [2.20*10^−3^–0.09], *P* = 1.22*10^−4^). Resampling also significantly increases the stability of and textural features in T1‐weighted images (median increase 0.14 [0.01–0.28], *P* = 1.87*10^−7^) but not in T2‐weighted images (median increase 0.01 [−0.11 to 0.18], *P* = 0.17).

#### 2.A.3. Stability to random noise

**Figure 3:**
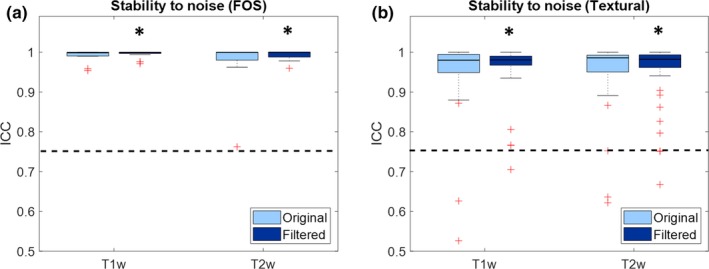
Boxplot representing the intraclass correlation coefficient (ICC) of the radiomic features for stability to image noise: (a) First order statistics; (b) textural features. Significant increase in ICC due to preprocessing are reported with asterisks. The dashed line represents the threshold of stability (ICC = 0.75). [Color figure can be viewed at http://wileyonlinelibrary.com]

Figure 3 shows boxplots representing the values of ICC of the features when the same phantom is acquired with different random noise. Values are shown for both T1‐weighted and T2‐weighted images, and for both original and gaussian filtered images. Only FOS and textural features are considered, since noise does not affect the shape and size features.

From Fig. 3 it can be seen that noise does not have much effect on the stability of radiomic features. In fact, most of the features are above the threshold of stability even before denoising. Gaussian filtering causes a small but significant increase in ICC for both FOS (T1w: median increase 7.76*10^−4^ [4.32*10^−6^–4.50*10^−3^], *P* = 1.96*10^−4^; T2w: median increase 1.84*10^−4^ [5.80*10^−6^–7.90*10^−3^], *P* = 3.26*10^−4^) and textural features (T1w: median increase 7.70*10^−3^ [5.17*10^−5^–0.03], *P* = 8.53*10^−6^; T2w: median increase 2.10*10^−3^ [−1.00*10^−3^–9.30*10^−3^], *P* = 0.03).

#### 2.A.4. Stability to intensity non‐uniformity

**Figure 4:**
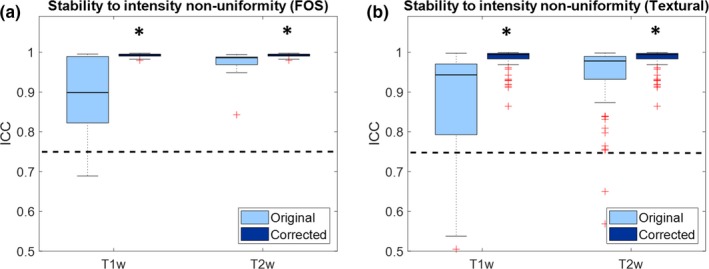
Boxplot representing the intraclass correlation coefficient (ICC) of the radiomic features for stability to intensity non‐uniformity: (a) First order statistics; (b) textural features. Significant increase in ICC due to preprocessing are reported with asterisk. The dashed line represents the threshold of stability (ICC = 0.75). [Color figure can be viewed at http://wileyonlinelibrary.com]

Figure 4 shows boxplots representing the values of ICC of the features when the same phantom is acquired with different intensity non‐uniformity fields. Values are shown for both T1‐weighted and T2‐weighted images, and for both original and bias field corrected images. Only FOS and textural features are considered, since intensity inhomogeneity does not affect the shape and size features.

From Fig. 4 it can be seen that the majority of features are above the threshold of stability. In both T1‐weigthed and T2‐weighted intensity standardization significantly increases ICC values of FOS features (T1w: median increase 0.09 [6.40*10^−3^–0.18], *P* = 5.35*10^−4^; T2w: median increase 7.40*10^−3^ [5.50*10^−3^−0.02], *P* = 1.96*10^−4^) and causes significant increase in ICC of textural features (T1w: median increase 0.05 [0.02–0.15], P = 3.38*10^−11^; T2w: median increase 0.01 [4.90*10^−3^–0.04], *P* = 2.48*10^−11^).

### 2.B. Stable features set identification

The features with ICC > 0.75 in all the previous analyses were included in the stable feature set. Table 2 shows the list of selected features, grouped by features class. In total, 67 stable features were identified for T1‐weighted images and 61 were identified for T2‐weighted images. As it can be seen from the Table 2, most of the stable features are stable in both the image types. The Jaccard index between the two features sets is 0.80.

**Table 2:** List of selected stable radiomic features grouped by features class and image type: shape and size, first order statistics (FOS), and textural. The red bold names refer to features that are not stable in the random parameter variations test.

Stable features				
Image type	Shape and size	FOS	Textural	
T1‐weighted and T2‐weighted	‐ Elongation ‐ Flatness ‐ Least axis length ‐ Major axis length ‐ Maximum two‐dimensional (2D) diameter (column) ‐ Maximum 2D diameter (row) ‐ Maximum 2D diameter (slice) ‐ Maximum three‐dimensional diameter ‐ Mesh volume ‐ Minor axis length ‐ Sphericity ‐ Surface area ‐ Surface to volume ratio ‐ Voxel volume	‐ 10th percentile ‐ 90th percentile ‐ Energy ‐ Entropy ‐ Kurtosis ‐ Maximum ‐ Mean ‐ Median ‐ Minimum ‐ Skewness ‐ Root mean squared ‐ Total energy ‐ Uniformity	‐ Autocorrelation ‐ Inverse difference ‐ Inverse difference moment ‐ Inverse difference normalized ‐ Joint average ‐ Joint energy ‐ Joint entropy ‐ Maximum probability ‐ Sum average ‐ Sum entropy ‐ Sum squares ‐ Gray level non‐uniformity (GLRLM) ‐ Gray level non‐uniformity normalized ‐ (GLRLM) ‐ High gray level run emphasis (GLRLM) ‐ Run length non‐uniformity ‐ Run length non‐uniformity normalized ‐ Run percentage ‐ Short run emphasis	‐ Short run high gray level emphasis ‐ Large area emphasis ‐ Zone percentage ‐ Zone variance ‐ Coarseness ‐ Strength ‐ Dependence non‐uniformity ‐ Dependence non‐uniformity normalized ‐ Dependence variance ‐ Gray level non‐uniformity (GLDM) ‐ High gray level emphasis (GLDM) ‐ Large dependence emphasis ‐ Large dependence low gray level emphasis ‐ Small dependence emphasis
T1‐weighted only			‐ Cluster shade ‐ Sum squares ‐ Long run low gray level emphasis ‐ Low gray level run emphasis ‐ Short run low gray level emphasis ‐ High gray level zone emphasis	‐ Large area high gray level emphasis ‐ Large dependence high gray level emphasis ‐ Low gray level emphasis (GLDM) ‐ Small dependence high gray level emphasis
T2‐weighted only		‐ Inter‐quartile range ‐ Mean absolute deviation ‐ Range ‐ Robust mean absolute deviation		

### 2.C. Robustness of stable features set on random simulations

After the stability analysis to random variations of the simulation parameters, most of the stable radiomic features were confirmed in both T1‐weighted and T2‐weighted images.

In the T1‐weighted images, 60 out of the 67 stable features (89.55%) were confirmed. Excluded features were the following: *maximum* (FOS); *maximum probability* and *sum square*s (GLCM); *gray level non‐uniformity normalized* and *long run low gray level emphasis* (GLRLM); *large dependence low gray level emphasis* (GLDM).

In T2‐weighted images, 51 out of the 61 stable features (83.61%) were confirmed. Excluded features were the following: *kurtosis*, *minimum* and *skewness* (FOS); *inverse difference*, *inverse difference moment* and *joint energy* (GLCM); *gray level non‐uniformity normalized* (GLRLM); *large dependence low gray level emphasis* and *small dependence emphasis* (GLDM).

## 3 Discussion

This work confirms that a non‐negligible percentage of radiomic features may vary considerably when image acquisition parameters are changed. Even when all preprocessing methods are applied), 46 out of 107 features (around 43%) had ICC < 0.75. It is fundamental to reduce the variability due to imaging settings and to define a set of stable features.

Variations in voxel size (PS and ST) represent the major source of variability especially for the textural features. This result is in line with previous studies performed on CT.^8^, ^10^ Variations in TR/TE reduce the reliability of the features, especially for FOS. However, for most of them the threshold of ICC remains above the threshold of stability. The same can be stated for variability caused by field strength inhomogeneity and image noise.

The optimal way to reduce image variability would be the standardization of the protocols for image acquisition. However, this is rather difficult when dealing with multi‐center studies and it is not an option when dealing with retrospective cases. Performing image preprocessing before any radiomic features extraction and defining a set of stable features seems to be the best way to standardize results in absence of a unique acquisition protocol. Resampling to a common resolution increases the reliability of the shape and size and textural features to variations of voxel size, even if some of them remain unreliable (ICC < 0.75). This is in line with previous phantom studies on CT.^8^, ^10^ Reliability of FOS features to variations of TR/TE can be significantly improved if intensity standardization is applied. Textural feature reliability is reduced by Z‐score normalization, but the effect, although significant, is very small. Preprocessing also helps increasing the stability to intensity non‐uniformities caused by field strength inhomogeneity. In particular, when the N4ITK algorithm is applied, stability is improved in both FOS and textural features. Last, image denoising through gaussian filtering causes small but significant improvements in stability of features to image noise.

Based on the results of the first four stability analysis two stable sets of features, one for T1‐weighted images and the other for T2‐weighted images, were identified. Over 80% of the stable features confirmed their stability when tested on MRI images simulated varying all the four parameters of interest, which of course is a further proof of the robustness of our feature sets. Moreover, many of the stable features were selected for both the image types (Jaccard index 0.80). This may suggest that there is a subset of MRI features whose stability is mainly independent on the image type considered. Further investigations may be performed to understand how the stable feature set that was identified in this study can be suitable for other types of MRI, such as diffusion‐weighted MRI or apparent diffusion coefficient maps. Based on our results, we may assume that those features are suitable for radiomic analysis of the brain using T1‐weighted and T2‐weighted MRI only.

The use of a virtual phantom helped in providing an easy and reproducible way to perform a large number of MRI simulations that were necessary to fully analyze the variability of radiomic features. This is not the first study in which MRI virtual phantoms are used.^11^, ^16^ In their experiment Ford et al.^11^ investigated variability of 29 radiomic features from T1‐weighted images due to changes in TR and TE with two univariate analysis. Although the metrics used are not the same (coefficient of variation instead of ICC) it is possible to notice that only a few radiomics features results as unstable (coefficient of variation > 30% as in defined in Shafiq‐ul‐Hassan et al.^10^), which is coherent with our findings. To the knowledge of the authors, our study is the most exhaustive in terms of variability analysis, because four different types of stability analyses were performed accounting for common sources of variability of the clinical practice.

This study is not exempt from limitations. One limitation is the fact that the custom MRI simulation was performed only on one phantom, in one district (the brain) and without considering pathological tissue. Additionally, other factors such as the type of scanner used, that are known to influence the radiomic features,^5^, ^8^ were not considered. These limitations can be partially addressed. Since many different tissues were considered for the stability analyses that were performed, we can assume that the results that were found in this study can be translated also to other district of the body and to pathological tissues, even though this assumption has to be verified in future studies. As far as the scanner variability and the inter‐phantom variability are concerned, those required necessarily studies on multiple real phantoms representing multiple districts. Such an extensive analysis is non‐trivial and requires much effort in the design of both the proper phantom and experiments to be done. To the extent of our knowledge, such an extensive analysis has not been done yet in any radiomic‐related studies. In fact, most of the phantom studies in literature involve only one phantom.^5^, ^8^, ^10^ Such a complex analysis is beyond the scope of this work but is definitely a goal for future ones. Both virtual and real phantoms may be useful for the assessment of reliability of radiomic features, since the former allows to better isolate the effects of the single sources of variability, while the latter allows more challenging and complete analysis that will be impossible otherwise. Therefore, the two types of analyses should be used complementarily rather than alternatively. However, experiment on virtual phantoms represent an easy way (sometimes the only feasible way) to obtain data that, although preliminary, may guide the selection of features for radiomic analysis.

## 4 Conclusions

In this study, stability of MRI‐radiomic features to image acquisition parameters was investigated for both T1‐weighted and T2‐weighted images. The effect of image preprocessing was evaluated and a set of stable features (ICC > 0.75) was evaluated for both the image types. The results of this study could provide knowledge to guide future studies on brain radiomics based on T1‐weighted and T2‐weighted MRI and to perform radiomic analyses even in those situations in which a protocol for image acquisition parameters does not exist.

## Conflict of interest

The authors have no conflict of interest.
